# Angioinvasive Scopulariopsis infection

**DOI:** 10.1016/j.jdcr.2024.10.024

**Published:** 2025-01-29

**Authors:** Meenakshi Manivannan, Damilola Oladinni, Vesna Petronic-Rosic, Joerg Albrecht

**Affiliations:** a Division of Dermatology, Department of Medicine, Cook County Health, Chicago, Illioins; b A.T. Still University School of Osteopathic Medicine in Arizona, Mesa, Arizona; c Department of Medicine, Rush Medical College, Chicago, Illinois

**Keywords:** invasive fungal infection, *Scopulariopsis* infection, tinea pedis

## Introduction

*Scopulariopsis* species (*spp*) are opportunistic fungi occasionally known to cause onychomycosis. Angioinvasive *Scopulariopsis* infection is a very rare but serious condition and the pathomechanism by which this fungus affects individuals is poorly understood. This case report highlights an infrequent manifestation of *Scopulariopsis spp* infection leading to angioinvasive disease.

## Case report

**Fig 1:**
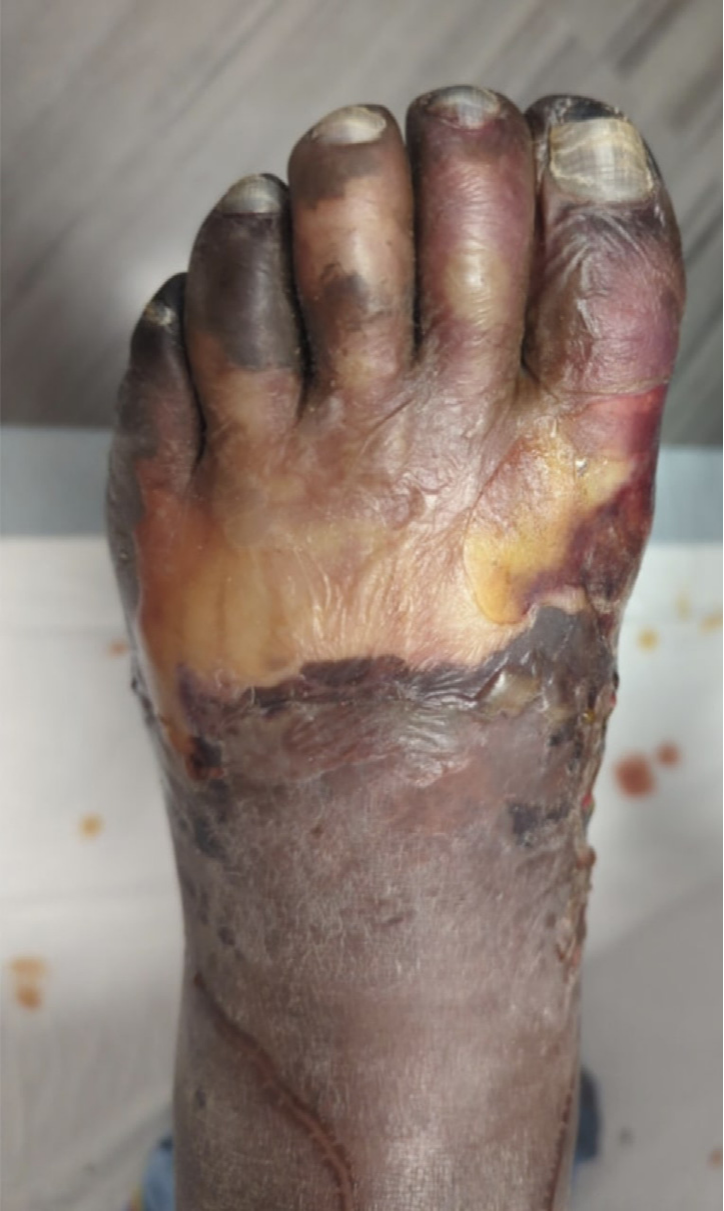
Well-demarcated violaceous and dusky discoloration of the distal foot with skin sloughing, fibrinous debris, and ischemic pallor (impending gangrene).

**Fig 2:**
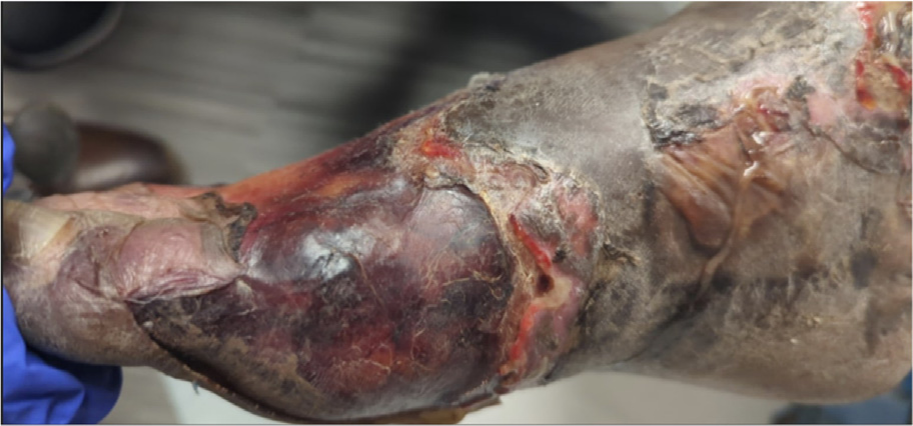
Progressive tissue necrosis of the distal foot.

A 49-year-old African American man presented to the dermatology clinic for evaluation of his feet. Initially, a slight violaceous discoloration affected the distal aspect of his toes (Fig 1). Over the course of 2 weeks, his symptoms progressed to well-demarcated violaceous - black discoloration, sloughing, and fibrinous deposits extending from the toes up to the mid-dorsal foot bilaterally (Fig 2). The distal toes were necrotic but not painful, probably due to longstanding peripheral neuropathy of both legs which caused general pain despite reduced peripheral sensation. The patient had a history of pyoderma gangrenosum (treated with infliximab infusions, cyclosporine oral solution, and clobetasol ointment) and Crohn’s disease with poorly controlled chronic pain. He followed up infrequently with the dermatology clinic and other specialists. Cyclosporine was recently restarted due to an exacerbation of the pyoderma gangrenosum. He took azathioprine intermittently to treat the Crohn’s disease.

**Fig 3:**
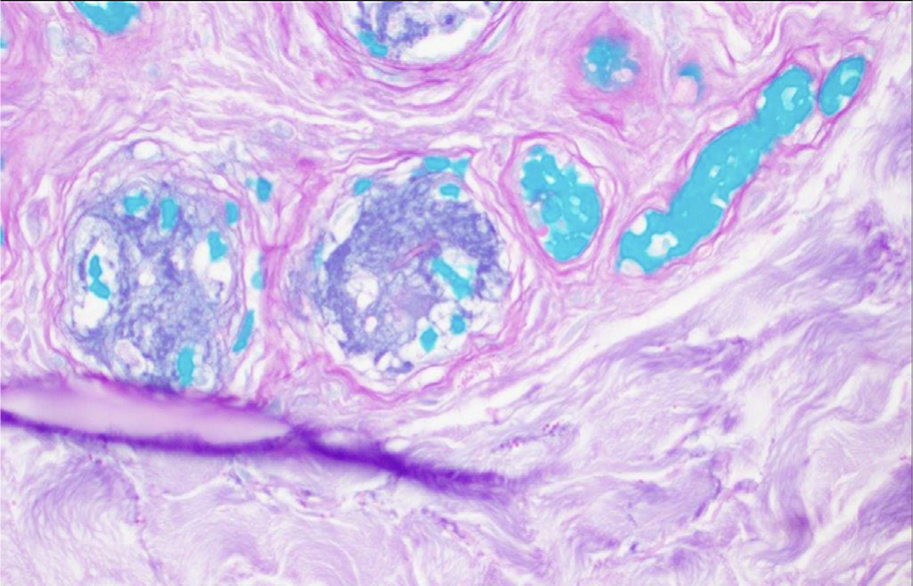
Intraluminal fibrin thrombi in the superficial dermal vasculature. Centrally, a PAS-positive hypha within the vessel lumen (PAS, 40×).

**Fig 4:**
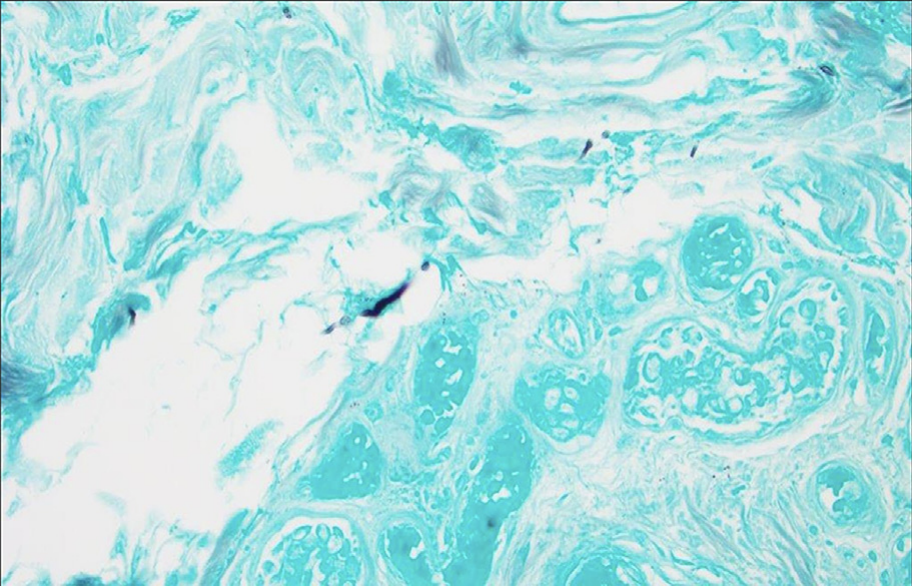
Broad ribbon-like hyphae are present throughout the dermis (GMS, 40×).

He was admitted to the hospital for further evaluation and possible vascular intervention. The clinical presentation was interpreted as probable frostbite by all other services. A couple of days later, a skin biopsy report showed scattered intraluminal fibrin thrombi involving the superficial dermal vasculature along with vascular dilatation, erythrocyte extravasation, and mild dermal edema. PAS and GMS stains revealed numerous broad ribbon-like hyphae throughout the tissue and within blood vessels (Figs 3 and 4). There were also gram-negative and gram-positive bacteria in the dermis. Tissue culture grew *Scopulariopsis species* and *Pseudomonas.* The patient was treated with intravenous ampicillin sodium/sulbactam sodium, amphotericin B, and caspofungin, while all his immunosuppressive medications were held. This regimen stopped the necrosis at midfoot. Nevertheless, he underwent bilateral below-knee amputation; which also removed some of the pain associated with his neuropathy. He is doing surprisingly well.

## Discussion

*Scopulariopsis spp* are rarely pathogenic saprobic, hyaline fungi found ubiquitously in the environment, particularly in soil, air, plant litter, paper, wood, dung, and animal remains.^1^ *Scopulariopsis spp* commonly causes noninvasive infections like onychomycosis, keratitis, and otomycosis. However, invasive disease, though rare, affects both immunocompetent and immunosuppressed individuals, manifesting in various forms, including endocarditis, sinusitis, brain abscess, deep cutaneous, localized pulmonary, and disseminated infections.^2^ It can be challenging to initiate aggressive and potentially toxic treatment quickly since the positive *Scopulariopsis* culture may be considered a contaminant, which they often are.^3^

A report by Steinbach *et al* stressed that despite efforts to combat *Scopulariopsis* infections, particularly in immunocompromised individuals, the limited therapeutic options and growing antifungal resistance pose significant challenges.^4^ The emergence of drug-resistant superinfections emphasizes the need for innovative management strategies and unique antifungal regimens. Many studies have suggested that *Scopulariopsis spp* are resistant *in vitro* to amphotericin B, flucytosine, and azole compounds.^4^ The literature suggests a rise in such fungal infections, urging clinicians to report experiences with newer therapies to enhance understanding and improve outcomes in vulnerable patient populations.

The concurrent identification of *Scopulariopsis species* and *Pseudomonas* in the patient’s tissue culture necessitated a comprehensive management approach. Given his immunocompromised status and limited antifungal options against *Scopulariopsis spp,* a multidisciplinary team collaborated to address both the infectious and inflammatory aspects. Despite not yielding optimal results in our patient, adjustments to the immunosuppressive regimen and timely antimicrobial therapy are crucial in maximizing optimal outcomes.

## Conflicts of interest

None disclosed.
